# CTAPIII/CXCL7: a novel biomarker for early diagnosis of lung cancer

**DOI:** 10.1002/cam4.1292

**Published:** 2018-01-22

**Authors:** Qiang Du, Encheng Li, Yonge Liu, Wenli Xie, Chun Huang, Jiaqi Song, Wei Zhang, Yijie Zheng, Huiling Wang, Qi Wang

**Affiliations:** ^1^ Department of Respiratory Medicine The Second Affiliated Hospital Dalian Medical University Dalian China; ^2^ Department of Respiratory Medicine The North Area of Suzhou Municipal Hospital Suzhou China; ^3^ Department of Clinical Laboratory The Second Affiliated Hospital Dalian Medical University Dalian China; ^4^ Department of Cardiology Medicine The Second Affiliated Hospital Dalian Medical University Dalian China; ^5^ Department of Health Statistics Second Military Medical University Shanghai China; ^6^ Department of Biostatistics School of Public Health Fudan University Shanghai China; ^7^ Medical Scientific Liaison Asian Pacific Abbott Diagnostics Division Abbott Laboratories Shanghai China

**Keywords:** Early diagnosis, lung cancer, serum biomarkers

## Abstract

It is desirable to have a biomarker which can facilitate low‐dose CT in diagnosis of early stage lung cancer. CTAPIII/CXCL7 is reported to be a potential biomarker for diagnosis of early lung cancer. In this study, we investigated the serum level of CTAPIII/CXCL7 in patients at different stage of lung cancer and the diagnostic efficacy of CTAPIII/CXCL7 in NSCLC. The plasma level of CTAPIII/CXCL7 was assayed by ELISA. CEA, SCCAg, and Cyfra211 were measured using a commercial chemiluminescent microparticle immunoassay. A total of 419 subjects were recruited, including 265 NSCLC patients and 154 healthy individuals. The subjects were randomly assigned to a training set and a test set. Receiver operating characteristic (ROC) and binary logistic regression analyses were conducted to evaluate the diagnostic efficacy and establish diagnostic mathematical model. Plasma CTAPIII/CXCL7 levels were significantly higher in NSCLC patients than in controls, which was independent of the stage of NSCLC. The diagnostic efficiency of CTAPIII/CXCL7 in NSCLC (training set: area under ROC curve (AUC) 0.806, 95% CI: 0.748–0.863; test set: AUC 0.773, 95% CI: 0.711–0.835) was greater than that of SCCAg, Cyfra21‐1, or CEA. The model combining CTAPIII/CXCL7 with CEA, SCCAg, and Cyfra21‐1 was more effective for NSCLC diagnosis than CTAPIII/CXCL7 alone. In addition, plasma level of CTAPIII/CXCL7 may contribute to the early diagnosis of NSCLC. CTAPIII/CXCL7 can be used as a plasma biomarker for the diagnosis of NSCLCs, particularly early stage lung cancer, with relatively high sensitivity and specificity.

## Introduction

Cancer is one of the most severe diseases that threaten human health, among which lung cancer is the leading cause of cancer death, accounting for about 19% of total cancer deaths 1. Lung cancer lacks typical symptoms at early stage. For this reason, most patients are diagnosed at an advanced stage, when effective treatment is limited. The 5‐year survival rate was 53.2% for localized lung cancer, but only 23.7% and 3.5% for advanced and metastatic diseases, respectively 2, 3. Therefore, early detection and treatment is essential to reduce lung cancer mortality.

A National Lung Screening Trial from American National Cancer Institute showed that low‐dose computed tomography (LDCT) based screening reduced the mortality of lung cancer by 20% 4. However, it brings harms as well as benefits, such as radiation effects, excessive cost, and overdiagnosis, which can be ascribed to the low specificity of LDCT 5. Swensen et al. 6 reported that less than 4% of the uncalcified pulmonary nodules were diagnosed as lung cancer. Hence, it is desirable to have a noninvasive test which can facilitate LDCT in diagnosis of early stage lung cancer.

CTAP III belongs to the subfamily of ELR^+^ CXC chemokines 7 that are potent regulators of angiogenesis 8, 9. It was reported to be a potential biomarker for diagnosis of lung cancer 10, 11. Stephen 10 reported that the elevation of this biomarker was detectable in stage 0/IA lung cancer. This is the first report that a blood biomarker can detect stage 0 lung cancer. It is reported that the AUC of receiver operating characteristic (ROC) curve was 0.64 (95% CI: 0.55–0.74) of CTAPIII/CXCL7 alone, but it can reach 0.81 (95% CI: 0.73–0.89) when CTAPIII/CXCL7 is combined with other clinical parameters, including age, smoking history, C‐reactive protein, and FEV1%. In 2011, Gina 11 drew a similar conclusion by mass spectrometry analysis of serum in lung cancer patients and high‐risk groups and reported that the AUC of CTAPIII/CXCL7 was 0.83. This suggests that CTAPIII/CXCL7 can be a potential biomarker useful for diagnosis of lung cancer.

In this study, we investigated the serum level of CTAPIII/CXCL7 in patients at different stage of lung cancer. We also examined the diagnostic efficacy of CTAPIII/CXCL7 in NSCLC and its diagnostic efficacy when combined with other biomarkers including CEA, SCCAg, and Cyfra211, which was recommended by National Academy of Clinical Biochemistry guidelines.

## Materials and Methods

### Patients and Samples

A total of 419 subjects were recruited from the Second Affiliated Hospital of Dalian Medical University, from October 2015 to June 2016, including 265 NSCLC patients and 154 controls (Table 1). The patients were treated in Department of Respiratory Medicine and Department of Thoracic Surgery, and the controls were from health checkup center. Lung cancer diagnosis was confirmed by pathology in all patients and staged according to the 7th IASLC/AJCC staging system 2. All lung cancer patients did not receive chemotherapy, radiotherapy, or surgery before blood sampling. All controls showed normal results in routine hematological test, liver biochemistry, renal function tests, electrolytes, abdominal ultrasound, and chest CT examination, and without any malignancy within 3 years. The study protocol was approved by the Ethics Committee of the Second Affiliated Hospital of Dalian Medical University.

**Table 1:** The characteristics of study subjects

	Training set			Test set		
	ADC	SCC	Controls	ADC	SCC	Controls
No. of patients	103	29	77	104	29	77
Age, years						
Media (range)	63 (35–85)	65 (49–80)	59 (34–85)	61 (34–82)	64 (47–84)	60 (34–79)
Sex						
Male/Female	37/66	23/6	44/33	42/62	27/2	42/35
No. of smokers	24	20	31	28	25	32
TNM stage						
Ia	44	4	–	37	2	–
Ib	6	0		15	2	
II	11	8	–	10	10	–
III	10	10	–	12	8	–
IV	32	7	–	30	7	–
T stage						
1	46	4	–	39	2	–
2	23	9	–	34	13	–
3	8	7	–	5	6	–
4	26	9	–	26	8	–
N stage						
0	64	13	–	59	12	–
1	9	3	–	9	6	–
2	9	8	–	13	4	–
3	21	5	–	23	7	–

ADC, adenocarcinoma; SCC, squamous cell carcinoma.

The patients and controls were randomly assigned to a training set and a test set. The training set comprised 209 subjects (132 NSCLC patients and 77 controls). The test set comprised 210 subjects (133 NSCLC patients and77 controls). The general clinical information is presented in Table 1, including age, sex, pathology, TNM stage. The training set was used to determine the cut‐off values for each biomarker and the diagnostic model. The test set was used to verify the diagnostic efficacy.

Blood samples were collected to EDTA tube before treatment and centrifuged at 1500*g* for 10 min immediately after collection (within 30 min) to separate serum, which was stored at −80°C until assay. The specimens were discarded if hemolysis or jaundice was identified.

### Biomarker assay

The levels of CTAPIII/CXCL7 were measured with a commercially available ELISA kit (ab100613, Abcam). All reagents were properly equilibrated to room temperature (18–25°C) prior to use. Add 100 *μ*L appropriately diluted specimen corresponding wells. The plates were covered well with sealer and incubated at room temperature (18–25°C) for 2.5 h with gentle shaking. The waste liquid was discarded. The plates were washed four times with washing solution. The liquid was completely removed after washing. Biotinylated CTAPIII/CXCL7 Detection Antibody (100 *μ*L) was added into each well. The plates were incubated at room temperature for 1 h with gentle shaking. After four rounds of washes with solution, 100 *μ*L of TMB One‐Step Substrate Reagent was added into each well and samples were incubated at room temperature for 30 min in the dark with gentle shaking. Finally, Stop Solution (50 *μ*L) was added to each well. The optical density was measured at 450 nm on SpectraMax 190 Microplate Reader (Molecular Device). The level of CTAPIII/CXCL7 was calculated with a quadratic polynomial fitting curve.

Other tumor markers, including CEA, SCCAg, and Cyfra211, were tested on an automated immunoassay analyzer, ARCHITECT *i*2000SR (Abbott Laboratories), using a commercial chemiluminescent microparticle immunoassay.

### Statistical analysis

When the concentration of analytes was measured in the sample, standard curve is calculated by Curve Expert (1.3 Edition). SPSS software package (version 21.0) was used to analyze data and Graph Pad Prism (version 5.0) was used to draw scatter plots. Considering the levels of CTAPIII/CXCL7 do not accord with normal distribution, the levels were expressed as median value followed by interquartile range (Q1,Q3). The level of CTAPIII/CXCL7 in different groups and stages were test by Kruskal–Wallis nonparametric test, and Nemenyi test was used as the post hoc test. Diagnostic efficacy was assessed by ROC curves. In order to find a cut‐off which is helpful for definitive diagnosis, the optimal cutoff value for each biomarker was determined when the specificity was 95% in the training set. A diagnostic model was built via binary logistic regression with the training set and was further assessed with the test set for the purpose to evaluate the diagnostic efficacy of biomarker combination. A two‐tailed *P *< 0.05 was considered significant.

## Results

### CTAPIII/CXCL7 level in NSCLC patients and controls

**Figure 1:**
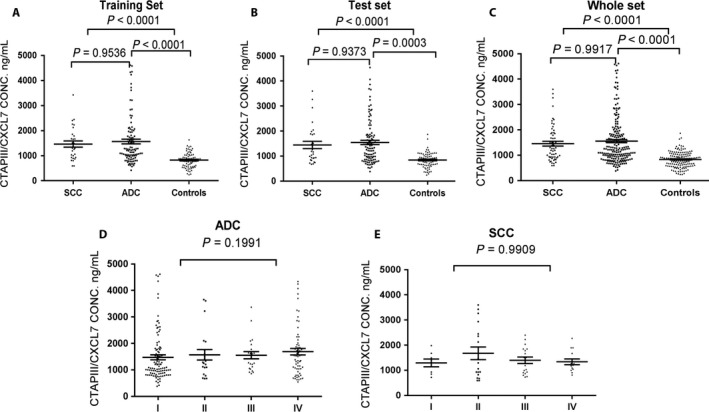
Plasma CTAPIII/CXCL7 concentration in lung cancer patients and healthy controls. Panels A, training set; B, test set; C, full set; D, patients with lung adenocarcinoma; E, patients with lung squamous cell carcinoma. The black horizontal lines indicate median values. *P* values were based on Kruskal–Wallis nonparametric test and Nemenyi test.

We compared CATPIII/CXCL7 level in the patients with squamous cell carcinoma, patients with adenocarcinoma, and controls. In the training set, the median level of CATPIII/CXCL7 was 1302.80 (931.84, 1790.14) ng/mL in the patients with squamous cell carcinoma and 1217.14 (970.40, 1940.93) ng/mL in the patients with adenocarcinoma, all significantly higher than that in controls (840.96 (585.53, 1023.92); Fig. 1A, *P *<* *0.0001). In the test set, the median level was1164.68 (913.17, 1883.89) ng/mL in the patients with squamous cell carcinoma and 1308.72 (864.51, 1835.60) ng/mL in the patients with adenocarcinoma, also significantly higher than that in controls (873.48 (667.61, 1022.08) ng/L; Fig. 1B, *P *<* *0.0001). When the two sets were combined, the median level of CATPIII/CXCL7 was 1258.99 (928.46, 1857.90) ng/mL in the patients with squamous cell carcinoma and 1257.03 (922.68, 1838.55) ng/mL in the patients with adenocarcinoma, all significantly higher than that in controls (851.80 (632.44, 1023.92) ng/L; Fig. 1C, *P *<* *0.0001). Generally, CATPIII/CXCL7 level did not show significant difference between the patients with squamous cell carcinoma and those with adenocarcinoma. However, plasma CTAPIII/CXCL7 level was higher in NSCLC patients than in controls.

### CTAPIII/CXCL7 level in terms of NSCLC disease stage

We analyzed CATPIII/CXCL7 level in terms of NSCLC disease stage. In patients with adenocarcinoma, CATPIII/CXCL7 was 1068.99 (862.81, 1785.81) ng/mL, 1284.59 (962.76, 1902.66) ng/mL, 1388.24 (1058.16, 1827.26) ng/mL, 1508.18(1001.12, 2035.68) ng/mL corresponding to disease stage I to IV, respectively. The highest CATPIII/CXCL7 level was observed in stage IV patients, but the difference between stages was not statistically significant (Fig. 1D, *P *>* *0.05). In patients with squamous cell carcinoma, CATPIII/CXCL7 was 1346.49 (869.74, 1632.24) ng/mL, 1176.80 (793.33, 2571.07) ng/mL, 1355.38 (903.20, 1856.07) ng/mL, 1222.86(1023.39, 1715.38) ng/mL corresponding to disease stage I to IV, respectively. The highest CATPIII/CXCL7 level was seen in stage II patients, but the difference between stages was not statistically significant (Fig. 1E, *P *>* *0.05).

### ROC analysis of CTAPIII/CXCL7, CEA, SCCAg, Cyfra211 and their combination

ROC curve was used to evaluate the efficacy of biomarkers (including CTAPIII/CXCL7, CEA, SCCAg, and Cyfra211) for diagnosing NSCLC. Table 2 summarizes the parameters related to diagnostic efficacy, including sensitivity, specificity, predictive value and likelihood ratio.

**Table 2:** The diagnostic efficacy of biomarkers in differentiating NSCLC patients and controls

	Training set							Test set						
	AUC (95% CI)	SN (%)	SP (%)	PPV (%)	NPV (%)	PositiveLR	NegativeLR	AUC (95% CI)	SN (%)	SP (%)	PPV (%)	NPV (%)	PositiveLR	NegativeLR
ADC vs. Controls														
CEA	0.711 (0.637–0.785)	41.7	95.0	91.8	54.9	8.34	0.61	0.737 (0.666–0.808)	35.6	96.1	94.9	52.8	7.12	0.66
SCCAg	0.669 (0.590–0.748)	15.5	95.0	80.6	45.7	3.10	0.89	0.634 (0.554–0.715)	19.2	94.8	86.9	46.8	3.84	0.84
Cyfra21‐1	0.679 (0.602–0.755)	32.0	95.0	89.5	51.1	6.40	0.72	0.646 (0.567–0.725)	30.8	96.1	91.4	50.7	6.16	0.72
CXCL7	0.800 (0.737–0.862)	41.7	95.0	91.8	54.9	8.34	0.61	0.775 (0.709–0.842)	49.0	96.1	94.4	58.2	12.56	0.531
SCC vs. Controls														
CEA	0.667 (0.546–0.787)	27.6	95.0	67.5	77.8	5.52	0.76	0.715 (0.618–0.813)	20.7	96.1	75.0	76.5	4.14	0.81
SCCAg	0.891 (0.824–0.959)	48.3	95.0	78.4	83.3	9.66	0.54	0.882 (0.808–0.957)	51.7	94.8	83.3	84.1	10.34	0.50
Cyfra21‐1	0.906 (0.836–0.975)	75.9	95.0	85.1	91.3	15.18	0.25	0.899 (0.820–0.978)	72.4	96.1	87.5	90.2	14.48	0.29
CXCL7	0.827 (0.736–0.918)	51.7	95.0	79.6	83.9	10.34	0.51	0.764 (0.661–0.867)	41.4	96.1	80.0	81.3	10.61	0.61
NSCLC vs. Controls														
CEA	0.701 (0.632–0.770)	39.4	95.0	93.1	47.8	7.88	0.64	0.732 (0.666–0.799)	28.6	96.1	97.4	44.5	5.72	0.72
SCCAg	0.718 (0.648–0.787)	22.7	95.0	88.6	41.8	4.54	0.81	0.688 (0.617–0.760)	26.3	94.8	92.1	43.0	5.26	0.77
Cyfra21‐1	0.729 (0.662–0.795)	40.9	95.0	93.3	48.4	8.18	0.62	0.701 (0.633–0.770)	39.8	96.1	94.6	48.0	7.96	0.63
CXCL7	0.806 (0.748–0.863)	43.2	95.0	93.7	49.4	8.66	0.60	0.773 (0.711–0.835)	45.9	96.1	95.3	50.7	11.77	0.56
Combination	0.909 (0.871–0.946)	72.7	95.0	96.2	67.2	14.58	0.29	0.892 (0.850–0.934)	62.4	96.1	96.5	59.7	16.00	0.39

LR, likelihood ratio; NPV, negative predictive value; PPV, positive predictive value.

**Figure 2:**
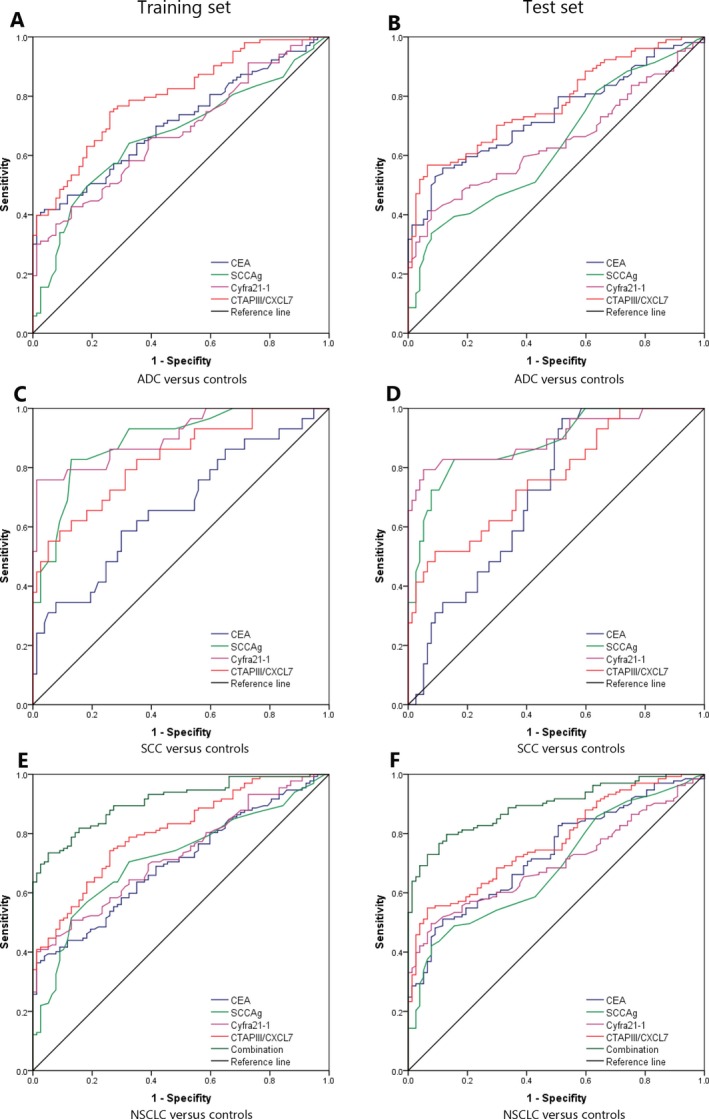
ROC curve analysis of CEA, SCCAg, Cyfra21‐1, CTAPIII/CXCL7, and biomarker combination in differentiating NSCLC patients and controls. Panels A&B: CEA, SCCAg, Cyfra21‐1 and CTAPIII/CXCL7 in patients with lung adenocarcinoma versus controls in the training (A) and test sets (B). Panels C&D: CEA, SCCAg, Cyfra21‐1 and CTAPIII/CXCL7 in patients with lung squamous cell carcinoma versus controls in the training (C) and test sets (D). Panels E&F: CEA, SCCAg, Cyfra21‐1, CTAPIII/CXCL7 and biomarker combination in NSCLC patients versus controls in the training (E) and test sets (F).

For lung adenocarcinoma, CTAPIII/CXCL7 showed the highest AUC (Fig. 2A, training set: AUC 0.800, 95% CI: 0.737–0.861; Fig. 2B test set AUC 0.775, 95% CI: 0.709–0.842) and satisfactorily separated the patients with lung adenocarcinoma from controls. The performance of SCCAg and Cyfra211 was relatively poor, with AUC ranging from 0.634 to 0.679. For lung squamous cell carcinoma, Cyfra211 showed the highest AUC (Fig. 2C, training set: AUC 0.906, 95% CI: 0.836–0.975; Fig. 2D test set AUC 0.899, 95% CI: 0.820–0.978) in distinguishing the patients with squamous cell carcinoma from controls. CEA showed lower discriminatory capacity, with AUC ranging from 0.667 to 0.715. For all the NSCLC patients combined, CTAPIII/CXCL7 exhibited optimal efficacy in diagnosis with the highest AUC (Fig. 2E, training set: AUC 0.806, 95% CI: 0.748–0.863; Fig. 2F test set AUC 0.773, 95% CI 0.711–0.835) (Table 2).

The optimal cut‐off value for each biomarker was selected according to ROC curve. Assuming 95% specificity in the training set, 1309.45 ng/mL was appropriate as the cut‐off value of CTAPIII/CXCL7 in NSCLC diagnosis (training set: sensitivity 42.3%, specificity 95%; test set: sensitivity 45.9%, specificity 96.1%). We selected 5.95 ng/mL as the cut‐off value of CEA in NSCLC diagnosis (training set: sensitivity 39.4%, specificity 95%; test set: sensitivity 28.6%, specificity 96.1%). As for SCC, 2.55 ng/mL was recommended as the cut‐off value in NSCLC diagnosis (training set: sensitivity 22.7%, specificity 95%; test set: sensitivity 26.3%, specificity 94.8%). The optimal cut‐off value of Cyfra211was 2.36 ng/mL in NSCLC diagnosis (training set: sensitivity 40.9%, specificity 95%; test set: sensitivity 39.8%, specificity 96.1%). In general, CTAPIII/CXCL7 showed better sensitivity than other biomarkers (Table 2).

A diagnostic model was built via binary logistic regression to investigate whether combining multiple biomarkers can yield better diagnostic efficacy. The combination of all biomarkers was expressed as:

$$\begin{array}{c} \text{Login(P)}=0.158\times \text{CEA}+0.863\times \text{SCCAg}+\\ 0.688\times \text{Cyfra211}+0.003\times \text{CXCL7}-6.056 \end{array}$$

According to ROC curve, the panel of multiple biomarkers in combination showed better discriminatory capacity (Fig. 2E, training set: AUC 0.909, 95% CI: 0.871–0.946; Fig. 2F test set: AUC 0.892, 95% CI: 0.850–0.934), significantly better than any single biomarker. In the training set, given 95.0% specificity, the sensitivity can reach 72.7%. At this risk coefficient, the specificity and sensitivity in the test set are 62.4% and 96.1%, respectively (Table 2).

### Diagnostic efficacy in early lung cancer

The patients with stage I or Ia lung cancer were selected and ROC curves were plotted to calculate the diagnostic efficacy of single or combined tumor markers in detecting early lung cancer (Table 3).

**Table 3:** The diagnostic efficacy of biomarkers in differentiating early stage NSCLC and controls

	Training set							Test set						
	AUC (95% CI)	SN (%)	SP (%)	PPV (%)	NPV (%)	PositiveLR	NegativeLR	AUC (95% CI)	SN (%)	SP (%)	PPV (%)	NPV (%)	PositiveLR	NegativeLR
Stage I vs. Controls														
CEA	0.472 (0.372–0.571)	1.9	95.0	21.0	58.0	0.38	1.03	0.556 (0.456–0.657)	7.1	96.1	57.0	58.7	1.82	0.97
SCCAg	0.630 (0.530–0.729)	1.9	95.0	21.0	58.0	0.38	1.03	0.614 (0.517–0.711)	14.3	94.8	66.7	60.3	2.75	0.90
Cyfra21‐1	0.587 (0.488–0.686)	13.0	95.0	64.6	60.9	2.6	0.92	0.541 (0.437–0.645)	10.7	96.1	66.6	59.7	2.74	0.93
CXCL7	0.778 (0.698–0.858)	35.2	95.0	83.2	67.6	7.04	0.68	0.729 (0.607–0.787)	39.3	96.1	88.0	68.5	10.08	0.63
Combination	0.794 (0.716–0.871)	44.4	95.0	86.2	70.9	8.88	0.59	0.804 (0.727–0.881)	42.9	96.1	88.9	69.8	11.00	0.59
Stage Ia vs. Controls														
CEA	0.453 (0.352–0.555)	2.1	95.0	20.7	60.9	0.42	1.03	0.522 (0.411–0.633)	0.0	96.1	0	65.5	0	1.04
SCCAg	0.619 (0.515–0.722)	2.1	95.0	20.7	60.9	0.42	1.03	0.598 (0.486–0.710)	12.8	94.8	55.5	68.2	2.46	0.92
Cyfra21‐1	0.558 (0.455–0.661)	8.3	95.0	50.9	62.4	1.66	0.97	0.525 (0.405–0.645)	7.7	96.1	50.0	67.3	1.97	0.96
CTAPIII/CXCL7	0.787 (0.706–0.867)	33.3	95.0	80.6	69.6	6.66	0.70	0.742 (0.644–0.840)	35.9	96.1	82.3	74.7	9.21	0.67
Combination	0.790 (0.708–0.872)	37.5	95.0	82.4	70.9	7.50	0.66	0.791 (0.700–0.881)	38.5	96.1	83.3	75.5	9.87	0.64

LR, likelihood ratio; NPV, negative predictive value; PPV, positive predictive value.

**Figure 3:**
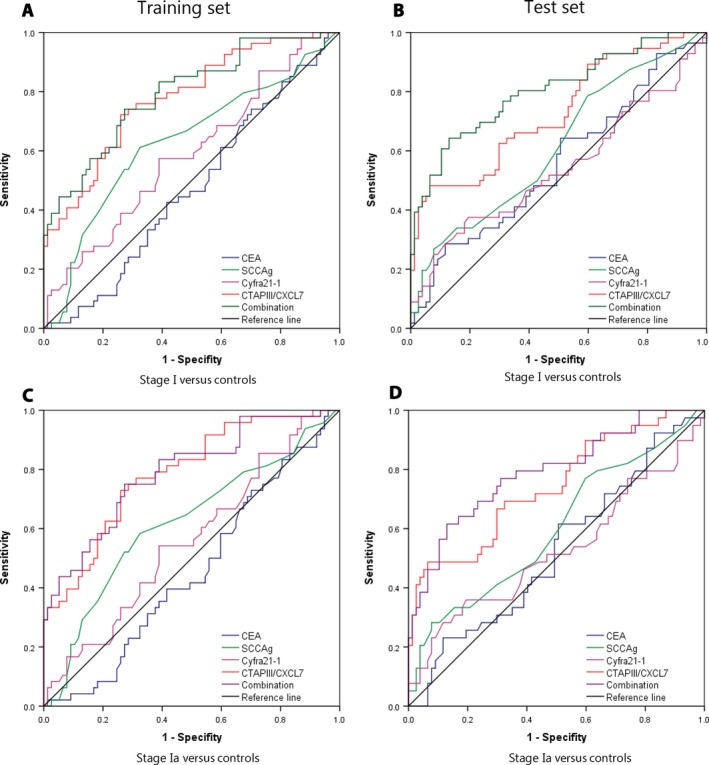
ROC curve analysis of CEA, SCCAg, Cyfra21‐1, CTAPIII/CXCL7, and biomarker combination in differentiating early stage NSCLC and controls. Panels A&B: CEA, SCCAg, Cyfra21‐1, CTAPIII/CXCL7, and biomarker combination in stage I NSCLC patients versus controls in the training (A) and test sets (B). Panels C&D: CEA, SCCAg, Cyfra21‐1, CTAPIII/CXCL7, and biomarker combination in stage Ia NSCLC patients versus controls in the training (C) and test sets (D).

For patients with stage I lung cancer, CTAPIII/CXCL7 had the highest AUC (Fig. 3A, training set: AUC 0.778, 95% CI: 0.698–0.858; Fig. 3B test set: AUC 0.729, 95% CI: 0.607–0.787). In the training set, given 95% specificity, the sensitivity is 35.2%, while in the test set, the sensitivity and specificity are 35.2% and 96.1%, respectively. When combined with other biomarkers, the sensitivity in the training set and test set can increase to 44.4% and 42.9%, respectively. It is evident that CTAPIII/CXCL7 has provided better diagnostic efficacy than other biomarkers (Table 3).

Similar results were observed in the patients with stage Ia lung cancer. Of the biomarkers tested, CTAPIII/CXCL7 had the highest AUC (Fig. 3C, training set: AUC 0.778, 95% CI: 0.706–0.867; Fig. 3D test set AUC 0.742, 95% CI: 0.644–0.840). In the training set, given 95% specificity, the sensitivity is 33.3%, while in the test set, the sensitivity and specificity are 35.9% and 96.1%, respectively. When combined with other biomarkers, the sensitivity in the training set and test set can be improved to 37.5% and 38.5%, respectively (Table 3).

So CTAPIII/CXCL7 performed better than other biomarkers in differentiating early stage lung cancer from healthy people.

## Discussion

Lung cancer is a common malignant tumor accounting for more cancer deaths than any other malignancies. It has been one of most serious condition threatening human health 1. Early detection and complete tumor resection would most likely provide a chance of cure for patients with early stage lung cancer. However, most patients were not diagnosed until advanced stage due to the asymptomatic nature of lung cancer. LDCT is helpful for screening and early diagnosis, but its usefulness is limited because of low specificity 5. In this study, we introduce a new biomarker which greatly improves the utility of LDCT in early diagnosis of lung cancer.

CXC chemokines have been implicated in various biological processes, including angiogenesis, anti‐angiogenesis, tumorigenesis, and metastasis, which are divided into two classes, ELR+ and ELR‐, based on whether they have specific amino acid sequence (ELR, Glu‐Leu‐Arg) 12. CTAPIII/CXCL7 is one of the most important members of angiogenic ELR+ CXC chemokine family 8, which is reportedly produced and stored in platelets, megakaryocytes, monocytes, lymphocytes and neutrophils 13, 14. After its release, CTAPIII can be cleaved by the proteases to CXCL7 15, 16, also known as NAP‐2, which can play its physiological role by binding to chemokine receptor CXCR1/CXCR2 through activation of the Ras/Raf/mitogen‐activated protein kinase (MAPK) and PI3K/AKT/mTOR signaling pathways 9, 17, 18, 19. Previous studies have shown that CTAP/CXCL7 is the invasion, metastasis, prognosis and diagnosis of some malignancies, including breast cancers 20, 21, 22, 23, malignant pancreatic diseases 24, 25, clear cell renal cell carcinoma (ccRCC) 26, colon cancer 27, and lung cancer 10, 11, 28. In breast cancer, CXCL7 can promote tumor metastasis by increasing expression of lymph angiogenesis factors VEGF‐C/D and heparanase 21. CXCL7 can also accelerate ccRCC development through binding CXCR1/CXCR2 to induce active of ERK pathway 26. In recent years, more studies report that CXCL7 is related to tumor diagnosis, such as pancreatic cancer 25, ovarian cancer 29, 30 and lung cancer 10, 11. Most interestingly, CXCL7 decreases in pancreatic cancer and ovarian cancer, but increases in lung cancer. It is reported that the elevation can be detected 29 months before the diagnosis of lung cancer.

We randomly assigned the subjects to training set and test set for the purpose to ensure the reliability and repeatability of our study. The cut‐off values and diagnostic model were validated with the training set, and the diagnostic efficacy was further verified with the test set. Our results indicate that plasma level of CTAPIII/CXCL‐7 is significantly higher in the patients with squamous cell carcinoma and adenocarcinoma than in controls. However, CTAPIII/CXCL‐7 did not show significant difference between the patients with lung adenocarcinoma and those with lung squamous cell carcinoma. This supports the usefulness of CTAPIII/CXCL‐7 in differentiating NSCLC patients from healthy people, but it is no use in identifying the pathological type of NSCLC. Furthermore analysis did not find significant difference of CTAPIII/CXCL‐7 level between different stages of lung cancer, which is unlike other traditional biomarkers 31, 32. The plasma level of CTAPIII/CXCL‐7 was independent of disease stage. Its elevation in stage I lung cancer is favorable for early diagnosis of lung cancer.

Considering the heterogeneity of NSCLC, some good tumor markers for the diagnosis of NSCLC were also introduced in this study, including CEA, SCCAg, and Cyfra211. SCCAg and Cyfra211 are useful for the diagnosis of squamous cell carcinoma and CEA is useful for the diagnosis of adenocarcinoma 31, 32, 33, 34. As it turns out, Cyfra211 showed higher sensitivity (72.4–75.9%) in diagnosis of squamous cell carcinoma than SCCAg, CEA, and CTAPIII/CXCL‐7, consistent with previous reports. In contrast, for diagnosis of lung adenocarcinoma, CTAPIII/CXCL‐7 provided higher sensitivity (41.7–49.0%) than CEA, SCCAg, and Cyfra211. For all NSCLC patients combined, CTAPIII/CXCL‐7 has the best diagnostic performance with sensitivity ranging from 43.2% to 45.9%. However, the sensitivity of any single biomarker is only about 22.7–40%, which apparently cannot satisfy clinical requirements. A predictive risk model was built via binary logistic regression. The results showed that the biomarker panel including CEA, SCCAg, Cyfra211 and CTAPIII/CXCL‐7 had significantly higher diagnostic efficacy than any single biomarker. The diagnostic sensitivity can be improved to 72.7% for NSCLC in the training set. These results were further validated in the test set.

Early diagnosis and prompt treatment is the most effective way to reduce mortality of lung cancer. The diagnostic performance of traditional tumor markers, including CEA, SCCAg and Cyfra211, is correlated with stage of tumor 31, 32, and their diagnostic efficacy is poor for early stage lung cancer, with low sensitivity for stage I NSCLC (1.9–14.3%). Our results showed that the level of CTAPIII/CXCL‐7 is not associated with the TNM stage of NSCLC. CTAPIII/CXCL‐7 performed better in diagnosis of stage I and stage Ia NSCLC than traditional markers, evidenced by significantly higher sensitivity (33.3–42.9%). Currently, LDCT is recommended as a tool to screen lung cancer in high‐risk population, but low specificity restricts its usefulness. Only about 2.7% of the lesions were malignant. Hence, CTAPIII/CXCL‐7 may serve as test to assist LDCT screening, which can differentiate early stage lung cancer from healthy people.

We have two hypotheses for the elevated levels of CTAPIII/CXCL‐7 in blood, which need to be further validated. The increase in CTAPIII/CXCL‐7 in blood may be related to tumor microenvironment and inflammatory reaction. Liu Suling 22, 23 reported that breast cancer stem cells can secrete IL‐6, which can up‐regulate CXCL7 production by mesenchymal cell. Meanwhile, CXCL7 is a cytokine which in turn activate STAT3/NF‐*κ*B signaling, leading to self‐renewal of breast cancer stem cells and a positive feedback loop was formed. Previous studies have reported that IL‐6 levels are higher in lung cancer patients than in healthy people 35, therefore, CTAP III/CXCL‐7 may be derived from the secretion of mesenchymal cell. In addition, according to Stephen 10, the level of CTAPIII/CXCL‐7 can decrease after surgery, and tumor recurrence was observed in the patients whose CTAPIII/CXCL‐7 level did not decrease. This phenomenon suggests that CTAPIII/CXCL‐7 may be originated from the autocrine of lung cancer cells. It is also reported CTAPIII/CXCL‐7 is secreted in other tumors, such as clear cell renal carcinoma 26.

There are some limitations in our research. The number of squamous cell carcinoma is not enough in this study because of inadequate number of specimens. So further studies with larger sample size are required to confirm these findings, and verify whether CTAPIII/CXCL‐7 is a specific marker of lung cancer. We are planning to conduct more researches to further explore the ability of CTAPIII/CXCL‐7 in differentiating lung cancer from benign nodules, including infectious lesion and benign tumor. In addition, this study did not recruit enough patients and compare the diagnosis power between lung cancer and patients with signs or symptoms of lung cancer, but with a different diagnosis (no neoplastic diseases). This information will further explore the differential diagnosis value of CTAPIII/CXCL7. We will also investigate and elaborate the molecular mechanism of CTAPIII/CXCL‐7 elevation in lung cancer patients and its physiological effects in future studies.

In summary, this study indicates that CTAPIII/CXCL‐7 performs better than traditional biomarkers CEA, SCCAg, and Cyfra211 in the diagnostic of early stage lung cancer. However, the diagnostic utility of any single biomarker is limited, and a panel of biomarkers combined can greatly improve the diagnostic efficacy. CTAPIII/CXCL‐7 has the best diagnostic efficacy for stage I and Ia lung cancer, which is helpful for judging the nature of the lesions detected by LDCT screening. CTAPIII/CXCL‐7 assay in combination with LDCT screening is favorable for shortening the diagnostic cycle and increasing the accuracy of diagnosis.

## Conflict of Interest

Dr. Yijie Zheng is employee of Abbott Diagnostics. The other authors have no declaration.
